# Changes in proviral load (PVL) among patients with rapidly progressive HTLV-1-associated myelopathy/tropical spastic paraparesis (HAM/TSP) receiving empirical therapy

**DOI:** 10.1186/1742-4690-8-S1-A58

**Published:** 2011-06-06

**Authors:** Eduardo Gotuzzo, Carolina Alvarez, Elsa González, Martin A Tipismana, Giovanni M López, Kristien Verdonck, Daniel Clark

**Affiliations:** 1 Instituto de Medicina Tropical Alexander von Humboldt, Universidad Peruana Cayetano Heredia, Lima, Perú; 2 Facultad de Medicina, Universidad Peruana Cayetano Heredia, Lima, Perú; 3 Departamento de Enfermedades Infecciosas, Tropicales y Dermatología, Hospital Nacional Cayetano Heredia, Lima, Perú; 4 Institute of Tropical Medicine, Antwerp, Belgium; 5 Laboratorios de Investigación y Desarrollo (LID), Facultad de Ciencias y Filosofía, Universidad Peruana Cayetano Heredia, Lima, Perú

## Introduction

Previous studies suggest that HTLV-1 PVL remains constant over time. We compare PVL, at baseline and follow-up, in patients with rapidly progressive HAM/TSP who received empirical treatment.

## Methods

Rapidly progressive HAM/TSP was defined by patients’ incapacity to walk unaided within two years after symptoms’ onset. These cases are treated at our center with prednisone in combination with Lamivudine and/or Zidovudine at standard doses. PVL were performed by duplicate in PBMC of blood samples drawn before (baseline) and during treatment; values are reported in copies /10^4^ PBMC.

## Results

We evaluated 11 patients (6 women); their median age was 50 years-old, interquartile range [IQR] 20, with significant differences between men and women (medians: 56 and 36, respectively, p=0.02). Median HAM/TSP duration at diagnosis was 12 months (IQR 4). The median time (all in months) between the symptoms onset and the beginning of therapy was 14 (IQR: 9); between HAM/TSP onset and the baseline PVL, 14 (IQR: 13); between PVL determinations, 7 (IQR: 7). Median PVL values were 1183 (IQR: 1425) at baseline, and 1002 at follow-up (IQR: 1425). In the group of 7/11 patients where PVL decreased, differences between baseline and follow-up determinations were significant (p=0.01), with a median paired reduction of 362 (IQR: 1425). In the four cases where PVL increased, differences were not significant (p=0.06).

## Conclusions

In most of the cases here reported, PVL decreased significantly during empirical therapy. Clinical correlates and long-term evaluation of these findings demand controlled therapy trials.

